# Eph/Ephrin-Based Protein Complexes: The Importance of *cis* Interactions in Guiding Cellular Processes

**DOI:** 10.3389/fmolb.2021.809364

**Published:** 2022-01-13

**Authors:** Alessandra Cecchini, D. D. W. Cornelison

**Affiliations:** ^1^ Division of Biological Sciences, University of Missouri, Columbia, MO, United States; ^2^ Christopher S. Bond Life Sciences Center, University of Missouri, Columbia, MO, United States

**Keywords:** signaling, development, cancer, Eph, ephrin, in *cis*

## Abstract

Although intracellular signal transduction is generally represented as a linear process that transmits stimuli from the exterior of a cell to the interior via a transmembrane receptor, interactions with additional membrane-associated proteins are often critical to its success. These molecules play a pivotal role in mediating signaling via the formation of complexes in *cis* (within the same membrane) with primary effectors, particularly in the context of tumorigenesis. Such secondary effectors may act to promote successful signaling by mediating receptor-ligand binding, recruitment of molecular partners for the formation of multiprotein complexes, or differential signaling outcomes. One signaling family whose contact-mediated activity is frequently modulated by lateral interactions at the cell surface is Eph/ephrin (EphA and EphB receptor tyrosine kinases and their ligands ephrin-As and ephrin-Bs). Through heterotypic interactions in *cis*, these molecules can promote a diverse range of cellular activities, including some that are mutually exclusive (cell proliferation and cell differentiation, or adhesion and migration). Due to their broad expression in most tissues and their promiscuous binding within and across classes, the cellular response to Eph:ephrin interaction is highly variable between cell types and is dependent on the cellular context in which binding occurs. In this review, we will discuss interactions between molecules in *cis* at the cell membrane, with emphasis on their role in modulating Eph/ephrin signaling.

## 1 Introduction

**FIGURE 1:**
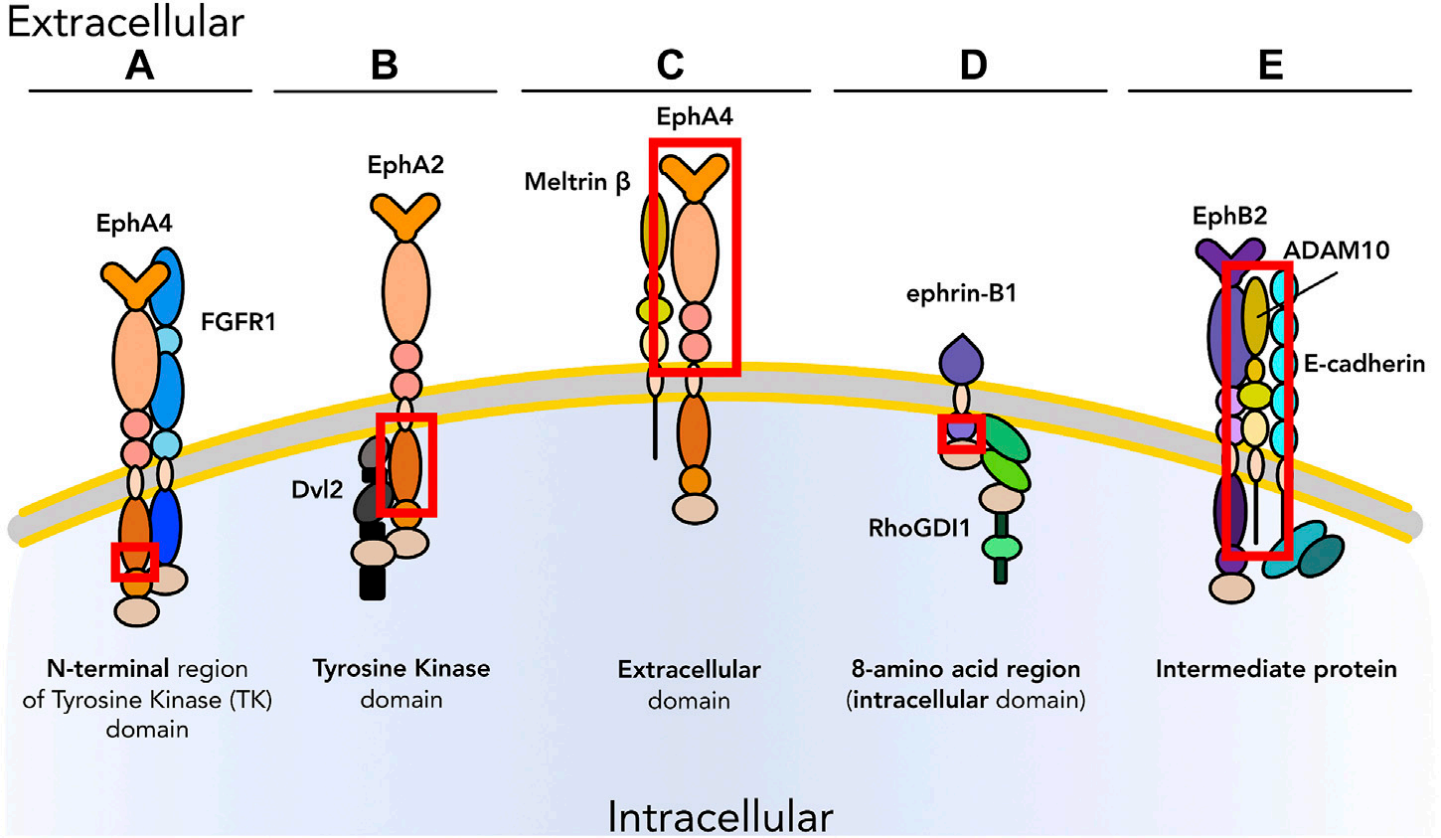
Graphical representation of examples of experimentally demonstrated in *cis* binding modalities of Ephs and ephrins with other membrane proteins. In *cis* interactions can occur via **(A)** N-terminal region of the tyrosine kinase (TK) domain; **(B)** TK domain; **(C)** extracellular domain; **(D)** intracellular domain (8-amino acid region); **(E)** intermediate proteins.

Cell identity and activity are regulated in large part by transduction of extracellular cues, the competence to receive and respond to which are dependent on the suites of membrane proteins, cytoplasmic effectors, and transcription factors expressed by the target cell. In this review, we will focus on the Eph/ephrin family of cell surface signaling molecules. Encoded by 14 unique genes, Ephs represent the largest class of mammalian receptor tyrosine kinases (RTKs): nine EphAs that interact primarily with five ephrin-As, and five EphBs that interact primarily with three ephrin-Bs (Pitulescu and Adams, 2010). Interactions between Ephs and ephrins mediate a broad variety of cellular pathways including proliferation, differentiation, cell adhesion, migration, survival, and apoptosis (Gucciardo et al., 2014). Binding is dependent on cell-cell contact, and Eph:ephrin interactions can signal either from the ephrin-bearing cell to the Eph-bearing cell (forward signaling), from the Eph-bearing cell to the ephrin-bearing cell (reverse signaling), or bidirectionally. In addition, unlike the majority of RTKs, Eph receptors and ephrin ligands may act both in *trans* (between two adjacent cells) and in *cis* (on the same cell) to promote or inhibit specific molecular cascades (Himanen et al., 2007; Arvanitis and Davy, 2008; Miao and Wang, 2012). Similarly, Ephs and ephrins directly interact with other membrane-associated signaling molecules in *cis* (Table 1) or in *trans*, increasing their repertoire of potential activities even further. Of particular interest for this review are interactions between molecules belonging to the ephrin family and other transmembrane proteins or cytosolic effectors recruited at the cell surface. Examples of experimentally demonstrated in *cis* binding modalities of Ephs and ephrins are shown in Figure 1 with the relevant domains highlighted. A) EphA4 binds FGFR1 though the N-terminal region of its tyrosine kinase (TK) domain: through FGFR1 pulldown assays with EphA4 deletion mutants a 126 amino acid (aa 636–762) region at the N-terminal of EphA4 TK domain was shown to bind the FGFR1 juxtamembrane domain (Yokote et al., 2005). B) EphA2 binds Dishevelled2 (Dvl2) via its TK domain: pull-down and co-IP experiments using mutants of EphA2 tagged with either GST or Flag showed the TK domain is crucial to binding of Dvl2 (Peng et al., 2018). C) EphA4 interacts with Meltrin β via its ectodomain: EphA4 co-immunoprecipitates with full-length Meltrin β but not with Meltrin β lacking its ectodomain (Yumoto et al., 2008). D) Ephrin-B1 interacts with RhoGDI1 via its intracellular region: deletion mutagenesis and immunoprecipitation revealed that amino acids 327–334 are critical for the binding of RhoGDI1 (Cho et al., 2018). E) Lastly, *cis* interactions may also be indirect via formation of a ternary complex. The transmembrane metalloprotease ADAM10 is required for physical interaction (demonstrated by co-IP) and activity of the complex of EphB2 and E-cadherin (Solanas et al., 2011) in epithelial cells. This review will address known and hypothesized *cis*-interactions at the cell membrane with emphasis on Ephs and ephrins, their role in regulating diverse cellular activities, and the potential for manipulation of these signals to generate novel targeting tools and treatments.

**TABLE 1:** Table summarizing the *cis* physical interactions between members of the ephrin family and signaling molecules involved in proliferation and differentiation, adhesion and migration, and cell signaling.

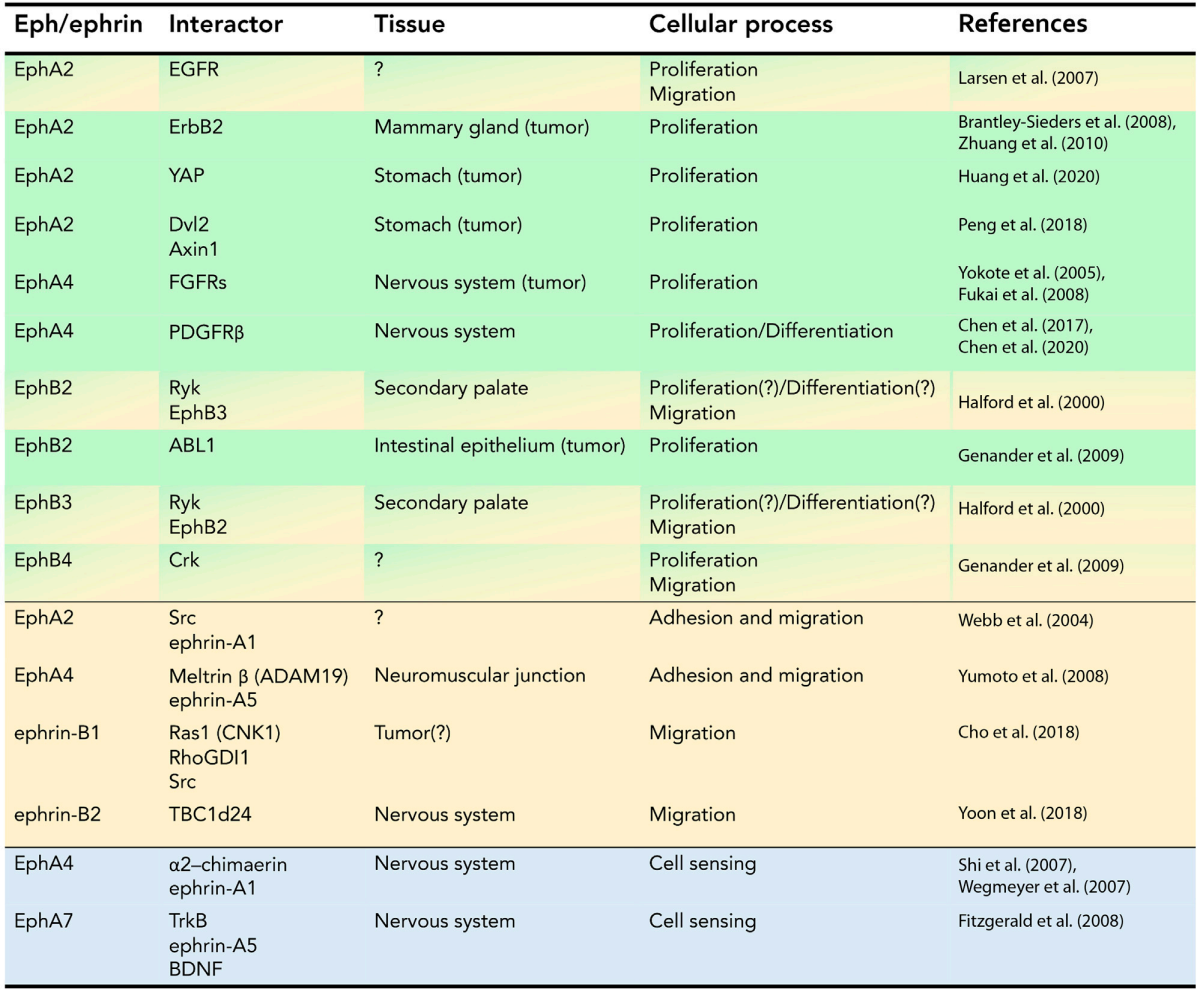

## 2 Cell Proliferation and Differentiation

**FIGURE 2:**
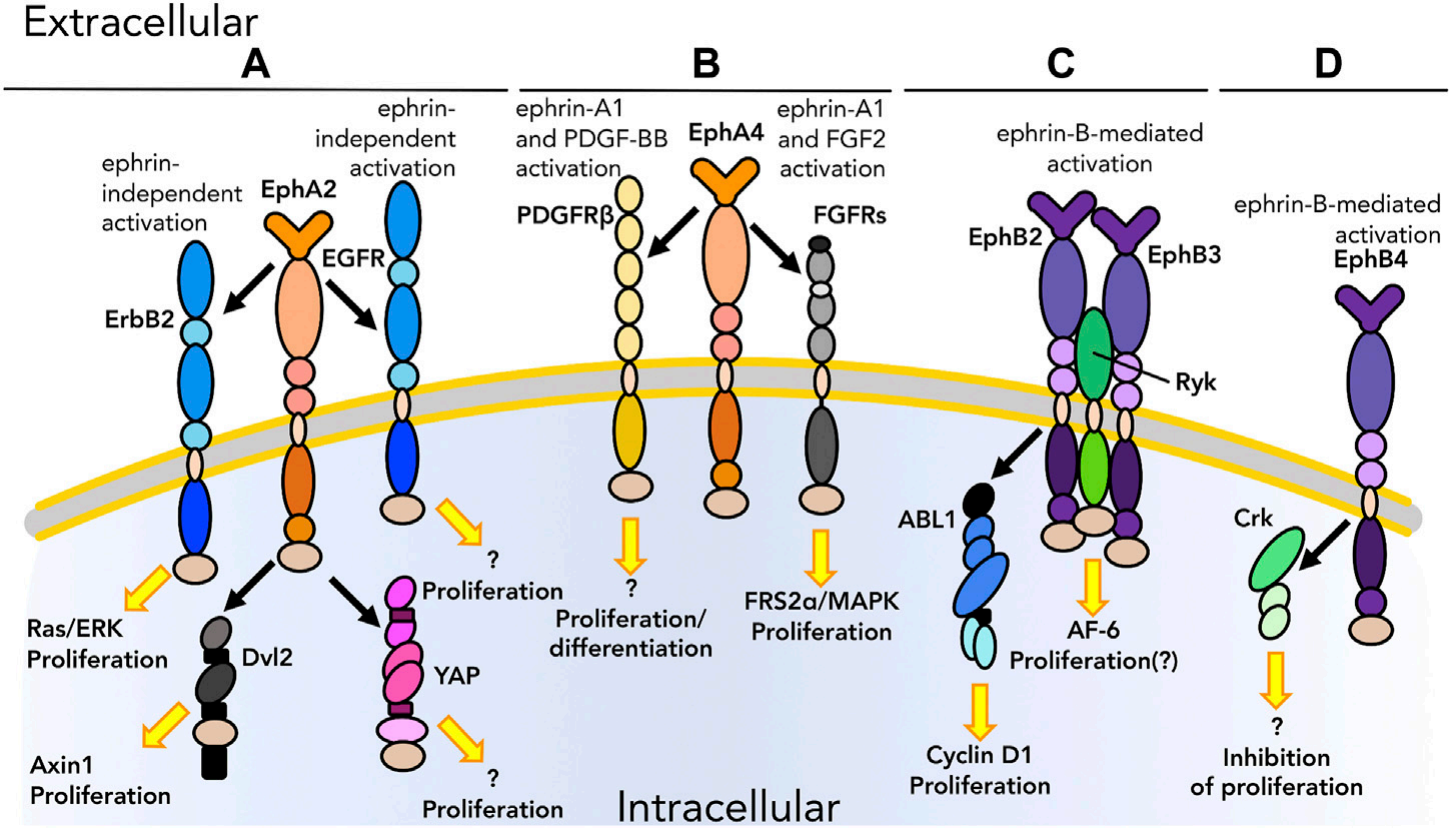
Graphical representation of physical interactions of ephrin receptors (Ephs) with other molecules to form heterotypic protein complex in *cis* that promote or inhibit cell proliferation and/or differentiation. **(A)** EphA2 interacts with EGFRs as well as cytoplasmic proteins, such as Dishevelled2 (Dvl2) and YAP, to promote proliferation in tumors in an ephrin independent manner. **(B)** EphA4 interacts with PDGFRβ and FGFRs upon ligand binding to regulate the balance between proliferation and differentiation in the nervous system. **(C)** Once activated by their ephrin ligand, EphB2 and EphB3 form a heterotypic multiprotein complex with Ryk during palate development in mice. EphB2 is also found to interact with ABL1; this interaction causes uncontrolled proliferation in the intestinal epithelium in the context of tumor. **(D)** Ephs can also impair proliferation, as in the case of the interaction between EphB4 and Crk. As we know only some of the downstream pathways, unknown downstream effectors are listed with a question mark. Receptors are depicted as single chains for simplicity. References are listed in Table 1.

Cell proliferation is a fundamental requirement for development, homeostasis, and response to injury. However, inappropriate or uncontrolled proliferation can be deadly: positive and negative regulation of proliferation in response to dynamic stimuli is a critical function of extracellular signaling. Often this regulation involves crosstalk and feedback between multiple signaling pathways, allowing integration of multiple signals into the decision to enter or exit the cell cycle. An example is the modulation of cell proliferation in olfactory epithelium neurons during development or after injury (Lander et al., 2009; Gokoffski et al., 2011) by feedback loops of activin (acting on stem and early progenitor cells) and GDF11 (acting on intermediate neuronal precursors) to maintain appropriate levels of differentiated cells. Similarly, cell differentiation is the stepwise process of specification and commitment leading to the generation of specialized cell types. In tissues such as in the skin (Gniadecki, 1998; Werner and Smola, 2001; Russo et al., 2020), differentiated cells are still able to proliferate to replace exhausted cells to renew the tissue and maintain its function. In other tissues such as skeletal muscle cell proliferation and differentiation are mutually exclusive processes, necessitating the involvement of stem cells (satellite cells in the skeletal muscle) to proliferate, commit, and generate post-mitotic terminally differentiated muscle cells that will fuse to form the functional unit of skeletal muscles, the myofiber (Linkhart et al., 1981; Olson et al., 1991; Shi and Garry, 2006). In these contexts Ephs and ephrins most often promote cell differentiation; one example is EphA7:ephrin-A5 interaction that guides *en masse* skeletal muscle cell differentiation during development and regeneration (Arnold et al., 2020). Table 1 (green section) and Figure 2 describe known physical interactions in *cis* between Ephs/ephrins and molecules regulating cell proliferation and differentiation.

### 2.1 The Effect of Cell Microenvironment on Cell Proliferation and Differentiation

An important contribution to the maintenance of this delicate balance comes from the surrounding environment. Extracellular stimuli such as growth factors and other soluble molecules, mechanical and oxidative stress, and homotypic and heterotypic cell-cell and cell-extracellular matrix (ECM) interactions are key for both preserving the stem cell pool and triggering the initiation of cell fate determination. Physical and biochemical cues are known to be able to modulate intracellular processes such as transcription. ECM in particular plays a central role: Connelly and collaborators (Connelly et al., 2010) reported the ability of differently patterned substrates to initiate differentiation in keratinocytes, thus demonstrating an effect of modifying the physical properties of the cell microenvironment, without any changes in its composition (i.e., chemical properties), on cell fate. Inputs from the local microenvironment can also be received by integrins and other adhesion molecules. β1 integrin-mediated adhesion, in combination with mitogen activated protein kinase (MAPK) signaling, can transduce extracellular stimuli from the surrounding environment and regulate transcription in epidermal stem cells (Guasch et al., 2007). Cell differentiation, comparable to cell proliferation, is therefore a spatio-temporal cellular mechanism guided by biochemical and mechanical stimuli and defined by a fine equilibrium with other cell behavior processes for the maintenance of the correct function of a tissue. Cell-cell contact and cell-microenvironment interactions are also factors guiding cell proliferation versus differentiation decisions. For instance, the unique microenvironment and architecture of many mesenchymal stem cell niches is sufficient to maintain cell stemness via controlling the rate and modality at which cells divide or undergo specification. Several groups have started focusing their attention on the role of physical forces in development, and in determining stem cell fate and cell differentiation. Mechanical forces as well as cell shape, ECM geometry and composition, and cell-cell and cell-ECM interactions also affect the balance of stemness-proliferation-differentiation (Clause et al., 2010).

Compromised expression and/or activity of proteins involved in interpreting the extracellular environment, including signaling receptors and adhesion molecules, can lead to an imbalance in proliferation versus differentiation, and in some cases abnormal outgrowth of cell masses and cancer. Cell-cell adhesion is modified in epithelial-mesenchymal transition (EMT), a phenomenon by which cells switch from an epithelial phenotype to a mesenchymal state and acquire the ability to migrate (which can contribute to metastasis and secondary tumor formation). The Twist/Snail family of TFs (Nieto, 2002), regulators of proliferation and stemness (Ignatius et al., 2017), is also tightly linked to EMT (Nieto et al., 1994). Snail1 can both induce the increase of tumor-propagating cells (Ignatius et al., 2017) and block differentiation, therefore maintaining an undifferentiated phenotype (Francí et al., 2006), and suppress epithelial cadherin (E-cadherin). Together these events lead to destabilized cell-cell interactions and favor EMT (Batlle et al., 2000). In the past decade, a few key studies have also highlighted the contribution of mesenchymal-epithelial transition (MET) to tumor aggressiveness. It has been shown that the success of metastasis (responsible for more than 90% of cancer associated mortality) depends on the re-aggregation of those tumor cells which underwent EMT in order to allow colonization of the secondary tumor site. Therefore, EMT is crucial for tumor cell dissemination and MET is key for efficient metastasis. In 2012, MET was experimentally demonstrated to have a role in the re-acquisition of not only a differentiated, epithelial phenotype, but also the ability of cells to proliferate by exiting their mesenchymal state, with the induction of Twist (Tsai et al., 2012).

### 2.2 Crosstalk Between Cell Proliferation and Differentiation Main Effectors and the Ephrin System

#### 2.2.1 FGF Receptors

The classical pathway to mediate cell proliferation is FGF. Critical to control progression of the cell cycle in multiple tissue types, FGFs and their receptors (FGFRs) are also overexpressed in many human solid tumors including breast, lung, gastric, and urothelial cancer (Katoh, 2019). The balance between cell proliferation and differentiation is regulated by FGFRs; signaling by FGFRs occurs through the activation of MAPK/extracellular signal-regulated kinase 1/2 (ERK 1/2) and phosphatidylinositol 3-kinase (PI3K)/protein kinase B (AKT) pathways, as well as the subsequent activation of signal transducer and activator of transcription3 (STAT3), phospholipase Cγ (PLCγ), and ribosomal protein S6 kinase 2 (RSK2) (reviewed in Turner and Grose, 2010 and Babina and Turner, 2017). Crosstalk between the FGF pathway and Eph/ephrin signaling modifies cell division and cell fate determination in both physiological contexts such as neurodevelopment (Picco et al., 2007) and pathological ones such as gastric cancer (Oki et al., 2008). FGFRs directly interact with EphA4 to promote cell proliferation in tumors such as glioma (Yokote et al., 2005; Fukai et al., 2008). EphA4 is also overexpressed in tumors such as breast and gastric cancer where FGFR levels are abnormally high (Oki et al., 2008; Dong et al., 2018). These membrane interactions in *cis* vary according to the identity of the FGFR: the juxtamembrane region of FGFR3 associates with the cytoplasmic domain of EphA4, while other FGFRs such as FGFR1 interact with EphA4 through the NH2-terminal region (Figures 1A, 2B) (Yokote et al., 2005). These close heterotypic associations result in the formation of a ternary complex of EphA4 and FGFRs that will promote cell proliferation and migration (Yokote et al., 2005) via the potentiation of FGF2 signaling in an ephrin-A1-mediated fashion, with phosphorylation of FRS2α and subsequent MAPK activation. Similarly, EphA4-FGFR1 signaling promotes cell proliferation and migration in human glioma by mutually amplifying their oncogenic potential (Fukai et al., 2008). While FGFRs are among the most studied receptors in cellular processes and human disorders, it may prove useful to expand these studies to include Ephs and/or ephrins as co-effectors that coordinate and boost tumor cell proliferation. Cancer is a heterogeneous disease and thus monotherapy via the targeting of a single molecule like FGFR is often not the ideal approach. Targeting of FGFRs for cancer therapy is well established and widely reviewed (Babina and Turner, 2017; Katoh, 2019; Xie et al., 2020; Krook et al., 2021), and there are also a few reports and patents describing successful targeting of exclusively EphA4 (Rajapakse et al., 2010; Dong et al., 2018), but dual or multi-molecule targeting strategies have not been explored yet.

#### 2.2.2 ErbB (EGF) Receptors

Uncontrolled cell proliferation associated with misregulation of EGFR is another well-described mechanism promoting cancer development and progression. EGFR is an RTK belonging to the family of ErbB receptors and it is overexpressed in the majority of human epithelial cancers; its activation upon ligand binding leads to enhanced cell division and inhibition of cell death, as well as activation of invasion potentiating pathways. Upon binding its ligand (commonly EGF or TGF-α) (Nicholson et al., 2001), a single-chain EGFR dimerizes to form an active homodimer or a heterodimer with EGFR, ErbB2, or ErbB4 (Lenferink et al., 1998), that stimulates intracellular pathways such as Ras/Raf (Buday and Downward, 1993; Li et al., 1993) and Src (Maa et al., 1995) to promote cell survival and proliferation (Scaltriti and Baselga, 2006). Ephs can form functional complexes with both ligand-bound and unliganded EGFR: co-staining for the two receptors showed co-localization at the plasma membrane, and their interaction has been confirmed by co-immunoprecipitation (IP) and proximity ligation assay (PLA) (Larsen et al., 2007; Swidergall et al., 2021), both with and without ligand stimulation. In the presence of EGF, the number of EGFR-EphA2 complexes in the membrane is increased and EphA2 is phosphorylated independently from ephrin stimulation (Figure 2A) (Larsen et al., 2007), leading to regulation of cell proliferation and motility. EphA2 has also been reported to compensate for the loss of EGFR signaling due to chemotherapy and thus maintain tumor progression (De Robertis et al., 2017). To our knowledge, only a couple of studies have been conducted to overcome EGFR therapy acquired resistance via EphA2 blockade (Amato et al., 2016; Kuo et al., 2020).

ErbB2 has also been reported to physically interact with EphA2 and promote an ephrin-independent response (Figure 2A). ErbB2 promotes cell survival and proliferation via activation of the PI3K/AKT pathway, when dimerization with other members of ErbB receptor family (e.g., ErbB3) occurs (Vivanco and Sawyers, 2002; Baselga and Swain, 2009). ErbB2 regulates cell proliferation in cancer (i.e., mammary adenocarcinoma) and its activity is further amplified by association in *cis* with EphA2 (Brantley-Sieders et al., 2008). These two receptors co-immunoprecipitate in primary tumor cells and appear to mutually affect proliferation of cancer *in vivo* (Brantley-Sieders et al., 2008). In the absence of EphA2, ErbB2-initiated tumorigenesis is decreased due to decreased Ras/ERK signaling, and inhibition of ErbB2 also inhibits EphA2 phosphorylation (Zhuang et al., 2010). This cooperation has been observed only in cancer (Brantley-Sieders et al., 2008; Zhuang et al., 2010) and other pathological conditions (e.g., pathogen infections) (Swidergall et al., 2021). It would be of interest to investigate if these physical interactions also occur under physiological conditions, potentially for the maintenance of homeostasis or wound healing, as another potential strategy for regeneration therapy.

#### 2.2.3 Wnt Signaling

The Wnt pathway is among the most ancient pathways involved in cell patterning and cell fate determination. Wnts can signal in a canonical manner via promotion of β-catenin-dependent transcription or through two β-catenin-independent, non-canonical pathways (Komiya and Habas, 2008). Wnt proteins bind the N-terminal cysteine-rich domain of Frizzled (Fz) transmembrane receptors, which physically interact with the cytoplasmic phosphoprotein Dishevelled (Dvl), a main downstream effector of the Wnt pathway. In the non-canonical planar cell polarity (PCP) pathway, Fz interacts in *cis* with co-receptors such as the catalytically inactive RTK Ryk (Lu et al., 2004) to modulate cell polarity (Andre et al., 2012), cell proliferation (Famili et al., 2016) and differentiation (Lyu et al., 2008). Ryk has been shown by co-IP experiments to interact with Ephs during secondary palate formation (Halford et al., 2000). In this context, Ryk interacts with EphB2 and EphB3 to form a complex (Figure 2C) and trigger Eph-dependent phosphorylation of Ryk. The assembly of this complex favors the interaction of Ryk with the downstream effector cell junction-associated protein AF-6 (Halford et al., 2000). Although Ryk has previously been associated with cell proliferation and differentiation, its interaction with EphBs has not been demonstrated to have a role in these processes.

Misregulation of the Wnt/β-catenin pathway can lead to uncontrolled cell division and tumor formation and growth. Aberrantly overexpressed EphA2 has been implicated in promoting EMT through Wnt/β-catenin signaling in cancer (Huang et al., 2017). EphA2 acts as a Wnt receptor by binding Dvl2 ligand via its TK domain (Figure 1B) (Peng et al., 2018) and recruiting Axin1 (Figure 2A), a scaffold protein that forms part of the cytoplasmic “β-catenin destruction complex”. If the formation of this complex is inhibited, β-catenin is not phosphorylated and thus targeted for degradation, and is able to translocate into the nucleus and activate transcription via Myc leading to uncontrolled proliferation and gastric cancer.

Unlike the interaction with other RTKs and receptors involved in the control of cell proliferation and differentiation, the crosstalk between Wnt signaling molecules and the ephrin system does not appear to occur via a mutual cross-activation. While the interaction of Ephs with FGFRs and ErbB receptors leads to cancer progression through synergistic activation of the receptors forming the complex, here Ephs act as direct negative regulators of the canonical Wnt pathway. Direct targeting of EphA2 and its subsequent inhibition in gastric cancer and other tumor types (e.g., colorectal cancer) has been already successfully achieved to control Wnt-dependent, EphA2-mediated tumor proliferation (Huang et al., 2017). Additional study of *cis* interactions at the plasma membrane and decoding where the two pathways interlock could pave the way to more translational interventions for digestive system tumors.

#### 2.2.4 Other Molecules Working in *cis* With the Ephrin System

Abelson protein tyrosine kinases (ABLs) are important players in controlling signaling pathways guiding cell growth and survival as well as adhesion, migration, and invasion. Depending on the direct interaction with a specific receptor and/or the production of mutated forms of ABL itself (e.g., v-ABL or Bcr-ABL), the ABL pathway can either promote tumor growth suppression or proliferation. For instance, in 2006, Noren and coworkers (Noren et al., 2006) reported inhibition of viability and proliferation and impaired motility and invasion through EphB4-mediated inactivation of the ABL-adaptor molecule Crk (Figure 2D). Similarly, in the intestinal epithelium EphB2 can promote cell proliferation via an ABL1-mediated increase in Cyclin D1 levels (Figure 2C) (Genander et al., 2009). The use of *in vivo* models, such as of *Abl1*-null mice, revealed a reduced number of proliferating cells even in the presence of active EphB2. Therefore, ABL1 was demonstrated to be required for EphB2-mediated cell proliferation in the context of intestinal adenoma.

Another example of interaction between Ephs and ephrins belonging to class A and other molecules playing a role in cell proliferation and differentiation is that of EphA4-PDGFRβ (Figure 2B). PDGFRβ is involved in development and homeostasis in multiple tissues including kidney, blood vessels, and the nervous system (Hellström et al., 1999; Nakagawa et al., 2011; Chen et al., 2020), as well as in cancer progression (Jitariu et al., 2018). In 2017, Chen and coworkers (Chen et al., 2017) demonstrated a direct interaction between EphA4 and PDGFRβ which leads to synergistic activation of proliferation of neural stem cells through the formation of a heterodimer capable of *trans*-autophosphorylation and subsequent activation of the molecular cascade. The same group later investigated the role of this interaction in neurogenesis in a mouse model of Alzheimer’s disease. This study demonstrated that the physical interaction between EphA4 and PDGFRβ promotes neurogenesis *in vivo* and proliferation and differentiation of neural progenitor cells *in vitro* upon ligand binding (ephrin-A1 and PDGF-BB, respectively) (Chen et al., 2020).

Finally, EphA2 has been shown to interact in *cis* with Yes-associated protein (YAP) (Figure 2A), a key molecule in the Hippo pathway and one of the main transcriptional coactivator molecules participating in homeostasis, cell proliferation, and stem cell maintenance. In gastric cancer, EphA2 interacts with and directly phosphorylates YAP, which causes YAP stabilization and its persistent presence in the nucleus. This novel EphA2-YAP interaction not only promotes gastric cancer growth but confers therapy resistance to these cells (Huang et al., 2020).

## 3 Cell Adhesion and Migration

### 3.1 What Is Cell Adhesion?

Cell adhesion is critical for both structure and function of organs and tissues, and regulates cell-cell and cell-microenvironment (i.e., ECM) communication as well as cell survival and function. Changes in cell adhesion are a hallmark of cancer progression and aggressiveness (i.e., metastasis). Cell-ECM adhesion is modulated by both molecular and mechanical forces, and in turn influences cell behavior and function. Via the fusion of materials science with biology, the nascent multidisciplinary field of mechanobiology has developed innovative tools for the study of cell adhesion. In this section, we will discuss cell-cell adhesion and cell-ECM adhesion via classical adhesion molecules and their contribution to cell adhesion and migration through the formation of functional heterotypic protein complexes.

#### 3.1.1 Cell-Cell Adhesion: Cell-Cell Communication in Healthy Tissues and Cancer

Classical cell adhesion molecules (CAMs) include the calcium-dependent cadherins and selectins as well as calcium-independent integrins, mucins, and immunoglobulin superfamily (IgSF). CAMs are transmembrane molecules in close contact with the cytoskeleton via their cytoplasmic domains. This facilitates transduction of biophysical cues to modify cell shape: for example, cadherins are known to signal via physical connections with filamentous actin (F-actin) (Acharya and Yap, 2016) through catenins (Maiden and Hardin, 2011). For cell-cell adhesion to occur, interaction in *trans* of these molecules has to be established by all contacting cells. However, co-effectors play a central role in facilitating this interaction and/or transducing the signal upon contact, through the formation of protein complexes in *cis*. Cadherins, selectins, and integrins are the main CAMs known to be involved in cancer progression. Cadherins play a major role in EMT and therefore tumor cell dissemination, whereas selectins and integrins mostly favor circulating tumor cell seeding at the secondary site by promoting interactions with local ECM components. Detailed roles of these CAMs in cancer have been reviewed by Läubli and Borsig (2019).

### 3.2 What Is Cell Migration?

Cell migration can be described as a cyclic process divided into four recurring phases: i) polarization of the cell and extension of protrusions, ii) formation of adhesion sites, iii) traction on the adhesion sites to accomplish movement, and iv) disassembly of adhesions. This articulate process requires complex interactions between suites of secreted and membrane bound molecules at each step including GTPases of the Rho family, focal adhesion kinase (FAK), cytoskeletal components, CAMs such as integrins, and ECM polymers. Cell migration can be a process accomplished by a single cell, for instance a fibroblast moving through the connective tissue, or collective migration such as spreading of epithelial sheets during wound healing and metastasis of tumor cells after EMT (Trepat et al., 2012; Li et al., 2013).

#### 3.2.1 Role of Cell Migration in Development

The initial step required for cell motility is cell polarity, which is tightly linked to cell adhesion. Cell polarity allows rearrangements in cytoskeletal actin and formation of cell protrusions to explore the surrounding environment and promote directional cell motility with the establishment of new adhesion sites at the interface cell-substrate. Small GTPases including RhoA are involved in these processes; for instance, RhoA has been shown to disrupt cell-cell adhesion and promote cell dispersion in Xenopus neural crest cells (Carmona-Fontaine et al., 2008). Rho regulates cell migration during primitive streak formation in mammalian embryos, and inhibition of Rho has dose-dependent effects on primitive streak formation (Stankova et al., 2015). Rho and other small G-proteins cooperate with CAMs (such as integrins) to promote cell adhesion and cell motility through the formation of focal complexes (Parsons et al., 2010; Lawson and Burridge, 2014). Other cells and/or the underlying matrix act as a patterned scaffold with a directionality that directs the orientation of cell migration. For instance, in addition to morphogen gradients, mesodermal cells are oriented by ECM fibrils on the blastocoel roof of amphibians (Nakatsuji and Johnson, 1984) and experimentally disrupting ECM organization leads to aberrant adhesion and migration during Xenopus embryogenesis (Rozario et al., 2009). Similarly, gonadotropin-releasing hormone neurons migrate towards the hypothalamus on ‘rails’ created by pre-existing aligned neural cells and cell projections (Cariboni et al., 2007).

#### 3.2.2 Cell Migration in Cancer Progression

The process of cell migration is also crucial for tumor progression and invasiveness. The role of chemoattractants (e.g., EGF) and surface receptors (e.g., EphA2) as driving molecules controlling cell adhesion and migration in cancer has been a focus of study for many years, and has been exploited for targeting of drug delivery and cancer treatment. The tumor microenvironment is complex and constantly evolving, and interactions between cells and ECM components are modified to promote cancer progression, as in the case of the interactions between macrophages and collagen and fibronectin in the ECM. While macrophages contribute to cancer progression mostly in a chemotactic manner (Wyckoff et al., 2004; Goswami et al., 2005), the crosstalk between ECM and cancer cells is purely based on adhesion signaling rather than paracrine signaling. Cytoskeletal rearrangement in response to chemotactic signals allows cancer cells to form actin-based structures, such as pseudopods and invadopodia, which sense the underlying matrix and promote migration forward or through the ECM respectively. The mechanisms at the basis of formation and action of pseudopods and invadopodia have been described in a recent review by Yamaguchi et al. (2005). Establishment of adhesion protein complexes such as focal complexes and focal adhesions (FAs) and activation of integrin pathways occur in parallel with the activation of other molecular cascades involving small G-proteins and intracellular kinases such as Rac1 and Ras (Yu et al., 2005), and Src (Frame, 2004) to modulate adhesion turnover and promote cell motility and tumor invasiveness.

### 3.3 What Are the Main Effectors Promoting Cell Adhesion and Migration?

#### 3.3.1 Cell Adhesion Molecules

CAMs are families of homotypic and heterotypic adhesion molecules that mediate the complementary processes of cell adhesion and cell motility. They can be divided into five classes: i) cadherins, ii) selectins, iii) integrins, iv) mucins, and v) IgSF CAMs, such as nectins. While cadherins and selectins act in a calcium-dependent manner, integrins, mucins, and IgSF are calcium-independent. These molecules can also be divided by those mediating cell-cell or cell-ECM adhesion: integrins are involved in cell-ECM adhesion, whereas cadherins, selectins, and IgSFs mediate cell-cell adhesion. Mucins fall into neither of these classes exclusively as their activity is mediated by interactions with other CAMs, such as selectins (Samanta and Almo, 2015; Harjunpää et al., 2019). CAMs are linked to the cytoskeleton, thus mediating cellular changes including cytoskeletal rearrangements and signal transduction in response to external stimuli. Different CAMs connect with different components of the cytoskeleton (e.g., cadherins are connected with cytoskeletal F-actin) and can promote adhesion by binding their counterparts on the adjacent cell or on the underlying substrate. These molecules are biological, dynamic bridges able to connect a cell either to its neighbors or to the surrounding ECM and to promote cell communication, sensing, and motility. To rapidly and appropriately sense physical cues that trigger the activation of mechanosensitive pathways, it is crucial for the cell to present a reactive, heterogeneous surface with a wide range of different adhesion molecules both to promote adhesion in dynamic contexts and to transduce different varieties of signals.

#### 3.3.2 Inside and Outside the Cell: Cytoskeleton and Non-Receptor Tyrosine Kinases and the ECM

The ECM has both passive and active roles in cell adhesion, as a substrate for integrin anchoring and as a source of mechanical stimuli or sequestered matrix metalloproteases (MMPs), respectively. The ECM is a dynamic support that connects cells with each other through multiple components, mostly proteoglycans and fibrous proteins (i.e., collagen, laminin, elastin). Depending on its composition, ECM will have different elasticity and stiffness, which are factors known to contribute to cell commitment and differentiation (Engler et al., 2006; Gilbert et al., 2010; Keung et al., 2011) as well as adhesion and migration (Pathak and Kumar, 2012; Hagiwara et al., 2021). MMPs, together with metalloprotease-disintegrins (ADAMs), guide ECM remodeling and are therefore involved in cell adhesion and cell adhesion-dependent processes (i.e., migration, proliferation and differentiation, apoptosis, polarization, gene expression) in both homeostasis and cancer. MMPs contribute to signaling pathways, such as cell adhesion and migration, through the interaction with other molecules and the formation of biologically active protein complexes. Examples will be found in Section 3.4.5.

The crosstalk between the adhesome and the cytoskeleton is a dynamic, bidirectional interaction: the formation and strengthening of adhesion sites is known to modulate cytoskeleton rearrangement, and the composition and architecture of the cytoskeleton regulates adhesion plaque turnover. This fluid relationship not only allows efficient cell adhesion but also coordinates cell migration (reviewed in Ridley et al., 2003). In addition to transmembrane proteins, cytoplasmic proteins such as non-receptor tyrosine kinases can tune adhesion and cell motility both positively and negatively by modifying the activity of signaling molecules. Src and ABL are two example molecules involved in multiple adhesion- and migration-related processes, from integrin function to turnover of cytoskeletal components. ABL is linked to F-actin when cells are in suspension, whereas when cells adhere ABL disassociates from F-actin (Woodring et al., 2001). Through this association, ABL is able to guide cytoskeletal rearrangements and cell adhesion and spreading by recruiting molecules belonging to the Rho family and Src (Woodring et al., 2002). Src is recruited to focal adhesions and activated upon integrin engagement; it is primed for activation by binding to the cytoplasmic domain of the integrin β-chain, and ligand-dependent integrin clustering leads to local accumulation of Src proteins and thus their *trans*-autophosphorylation. For a review of the role of Src as well as CAMs and actin modulators in cell adhesion, the reader is referred to Brunton et al. (2004).

#### 3.3.3 Mechanical Forces in Cell Adhesion and Migration

Mechanical cues play a crucial role in biology, especially in development and cancer. As mentioned above, cell adhesion and migration are biological and physical processes that involve physical contact-dependent activation of mechanosensitive molecules to trigger adhesion signaling cascades and cell motility. Adhesion molecules bind to a plethora of other primary or secondary adhesion effectors to form complexes acting as sensors for mechanical forces (Eyckmans et al., 2011). These protein complexes are classified as adhesive junctions (AJs) and desmosomes in cell-cell adhesion and FAs and hemidesmosomes in cell-substrate context. Cadherins are assembled into AJ followed by intracellular recruitment of catenins and vinculin. Full AJ maturation requires mechanical forces derived from the formation of actin stress fibers following cell binding to the ECM and accumulation of vinculin at the adhesion site in a catenin-dependent manner. Particularly, atomic force microscopy studies have shown the ability of catenins (i.e., α-catenin) to induce vinculin conformational changes and strengthen adhesion. In this way, catenins act as mechanosensors to promote AJ-mediated cell-cell adhesion in a force-dependent fashion (Yonemura et al., 2010; Maki et al., 2016).

Desmosomes have also been shown to be able to process mechanical stimuli via their linkage to cytoskeletal intermediate filaments (IFs) through cadherins. Through these connections, desmosomes are able to stabilize cell-cell adhesion, and because of their “hyper-adhesiveness,” they also provide cells with resistance to mechanical stress (Garrod and Chidgey, 2008), such as shear stress.

Full maturation of FAs requires stress fiber formation upon tension and contraction. Once a cell makes contact with the ECM via integrins, nascent FAs are formed, stress fibers are polymerized, and FAs complete their maturation in a force-dependent manner. The major components of FAs are talin and vinculin. Talin is well-established as a mechanosensitive molecule, and its role in mediating mechanotransduction has been discussed in detail in other reviews (Haining et al., 2016; Goult et al., 2018). Talin cooperates with other FA molecules (i.e., vinculin and integrins) in response to external stimuli, such as ECM flexibility and stiffness, and regulates the strength of cell adhesion. Myosin II plays a key role in adhesion maturation through talin-integrin and talin-vinculin interactions. The precise mechanism has been described in detail by Parsons and colleagues in their 2010 review (Parsons et al., 2010).

Hemidesmosomes, similarly to FAs, are involved in ECM stiffness sensing. Zhang et al. (2011) demonstrated the effect of tension on morphogenesis in *Caenorhabditis elegans*, via the ability of hemidesmosomes to act as mechanosensors. They demonstrated tension-dependent activation of hemidesmosomes, which leads to phosphorylation of IFs through the Rac pathway and epithelial morphogenesis in this model.

### 3.4 How the Ephrin System Interacts With Adhesion and Migration Key Proteins: Physical and Biochemical Interactions

**FIGURE 3:**
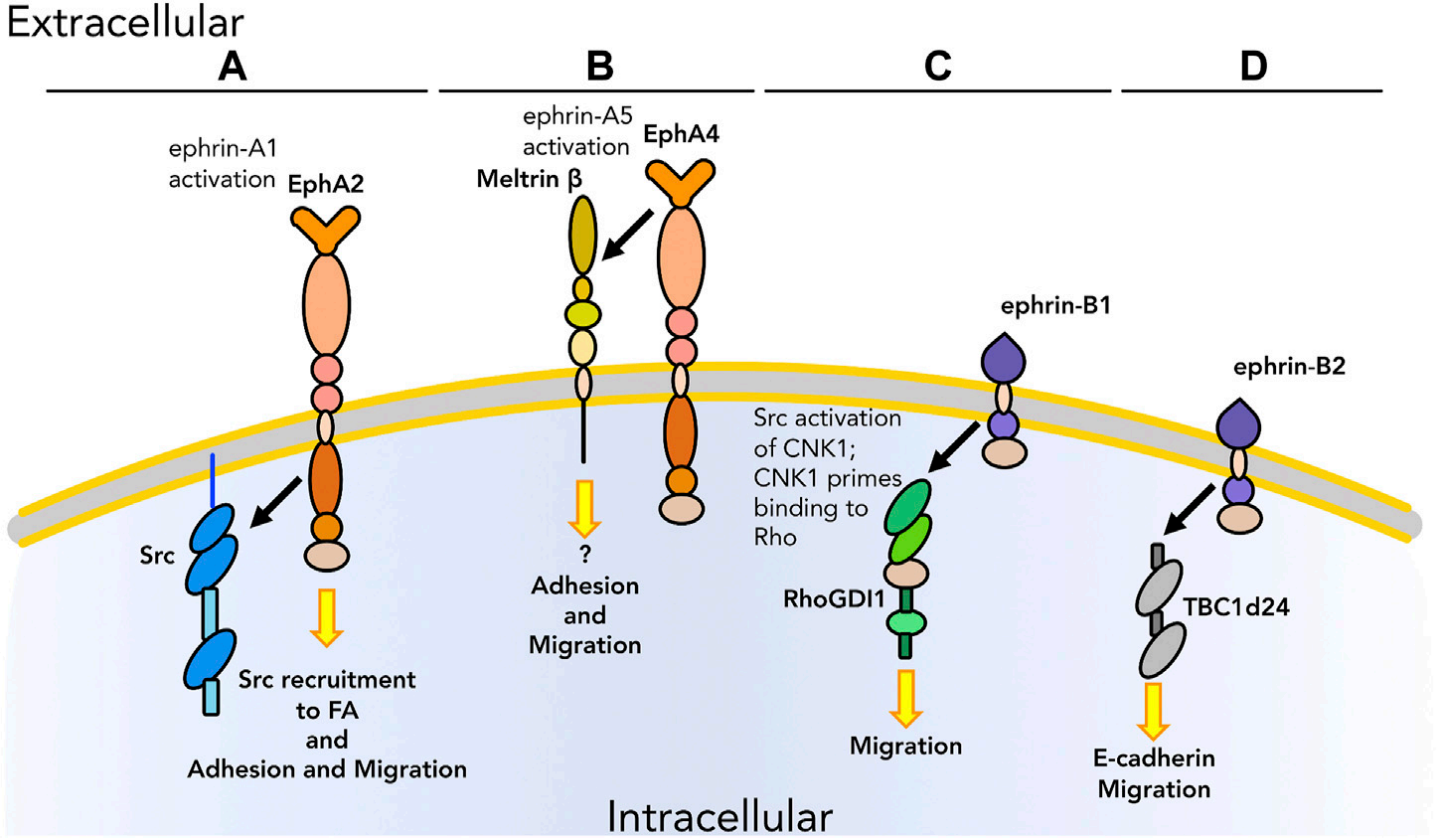
Graphical representation of physical interactions occurring in *cis* between Ephs/ephrins and other molecules to promote or inhibit cell adhesion and migration. **(A)** After activation by ephrin-A1 binding, EphA2 promotes the recruitment of Src at FA for the regulation of adhesion and migration. **(B)** EphA4 interacts with the ectodomain of meltrin β through its extracellular domain to regulate adhesion and migration at the NMJ. **(C)** ephrin-B1 interacts with RhoGDI1 to enhance migration in tumors. **(D)** ephrin-B2 interacts with TBC1d24 to modulate migration via E-cadherin in the nervous system. As we know only some of the downstream pathways, unknown downstream effectors are listed with a question mark. Receptors are depicted as single chains for simplicity. References are listed in Table 1.

A unique aspect of Eph:ephrin interactions lies in the requirement for two cells to be in direct contact for signaling to occur. Because the ligand ephrin is not secreted but membrane-anchored like its receptor, Ephs and ephrins are well-suited to mediate activities such as adhesion and cell motility. It would therefore not be surprising for Ephs and ephrins to interact as part of larger adhesion and signaling complexes. Indeed, crosstalk between the Eph/ephrin axis and cell adhesion effectors such as CAMs, MMPs, and cytoplasmic enzymes (e.g., Src) has been uncovered in multiple cellular contexts. This section will discuss these direct interactions between Ephs and ephrins and cell adhesion/migration pathways in healthy tissues and cancer (Table 1, yellow section; Figure 3).

#### 3.4.1 Integrins

Integrins are able to interact with a wide variety of ECM molecules, plasma membrane proteins, and secreted molecules. Integrins are membrane receptors composed of one α-chain and one β-chain. In humans, there are 18 types of α subunit and 8 types of β subunits; the different α/β composition allows integrins to interact with different ECM molecules and thus they direct substrate-specific adhesion and downstream signaling in multiple tissues under both physiological and pathological conditions (Bökel and Brown, 2002; Gahmberg et al., 2009; Hamidi and Ivaska, 2018). In neurodevelopment, integrin-mediated adhesion is often tuned by the ephrin system. An example is the ability of ephrin-A5 to improve cell adhesion to fibronectin and control cell morphology in an integrin-dependent fashion. Davy and Robbins demonstrated that the activation of EphA5-ephrin-A5 signaling modulates cell-ECM adhesion via the phosphorylation of integrin activation-associated downstream effectors, such as FAK, and cell morphology key molecules, such as MAPK. The activation of these pathways promotes changes in adhesion properties that result in neurite outgrowth and axon guidance in the nervous system (Davy and Robbins, 2000). In addition to EphA5-ephrin-A5 signaling, EphA2 has also been demonstrated to contribute to the regulation of adhesion to fibronectin in human dendritic cells (De Saint-Vis et al., 2003). Similarly, the activation of EphA4 upon binding of its ligand causes the inhibition of integrin activity and subsequent dendritic spine retraction in pyramidal neurons (Bourgin et al., 2007). In light of these findings, it is intriguing how different Eph/ephrin signaling pathways can promote two opposite effects in the same tissue, even when acting on the same molecular pathway (i.e., β1 integrin-mediated cell adhesion). Eph and ephrins are best characterized as promoting cell repulsion in the nervous system (Muñoz et al., 2005; Gallarda et al., 2008), therefore observation of dendritic retraction in this tissue is not unexpected. Conversely, the role of the ephrin system on neurite outgrowth and axon guidance can lead to new paths to explore in biosensor design and possibly nerve regeneration.

An example of integrin-Eph/ephrin crosstalk in a non-neural setting is that occurring between EphA2 and integrin α3. EphA2 interacts in *cis* with different molecules, from growth factors to adhesion molecules (listed in Table 1) to regulate cell adhesion and migration (Miao et al., 2000; Petty et al., 2012), especially in the context of cancer development and progression. Makarov et al. (2013) reported the co-localization of both EphA2 ligand (in this case, ephrin-A1) and integrin α3 at the cell membrane, and in particular, co-localization of EphA2 with integrin α3 at the protrusions and focal adhesion sites of U25MG1 glioblastoma cells to promote migration. Although the contribution of EphA2 to protein complexes mediating integrin α3-guided adhesion and migration was reported, the authors were not able to identify a direct interaction of EphA2 with integrin.

#### 3.4.2 Cadherins

Cadherins are type I integral membrane glycoproteins of about 750 amino acids, consisting of an extracellular domain, a single transmembrane spanning region, and an intracellular domain. Cadherins are the most abundant cell-cell adhesion molecules in solid tissues and are stabilized by calcium ions, hence their calcium-dependent function and the origin of their name: CAlcium Dependent adHERent proteINS (Patel et al., 2003). Although evidence of biochemical interaction between the ephrin system pathways and cadherin signaling has been widely reported, to date, no clear evidence of direct physical *cis* interaction between these two protein families has been demonstrated. Since early 2000s, localization of Eph/ephrin to cell-cell contact sites has been reported to be cadherin-guided and associated with changes in cell adhesion, but no Eph/ephrin-cadherin direct *cis* interaction was detected (Zantek et al., 1999). This crosstalk may also occur in the opposite direction: Eph/ephrins have been shown to modulate cadherin-mediated cell adhesion by acting on cadherin clustering at adhesion sites (Fagotto et al., 2013). Regulation of cadherin-mediated adhesion by the ephrin system can also occur indirectly via activation of surface proteins acting as coreceptors, as in the case of ADAM10 metalloprotease (Solanas et al., 2011) (Figure 1E).

It is intriguing that the literature contains multiple descriptions of crosstalk between Eph/ephrins and adhesion molecules such as integrins and cadherins, but almost no data on their physical interaction with each other. While the formation of complexes between integrins or cadherins and Ephs/ephrins would be expected in light of the mutual regulation of their pathways and close proximity in membrane domains, those interactions that have been described are entirely biochemical, acting exclusively through co-effectors and downstream molecules (e.g., ADAM10) participating in cell adhesion and migration. Further studies into this gap in our understanding will ideally lead to better understanding of the spatial association between Ephs/ephrins and adhesion molecules, as well as address the mechanics and dynamics of their interaction from a new perspective.

#### 3.4.3 Claudins

A class of adhesion molecules of particular interest for this review is the claudin family. Claudins are four-transmembrane domain proteins that play a central role in cell-cell communication. Together with occludins, they form tight junctions to regulate solute transportation at the apical region of polarized epithelial and vascular endothelial cells. Claudins are particularly important during embryonic development and morphogenesis, and diverse claudins are expressed in a spatio-temporally regulated manner during embryogenesis. Collins et al. (2013) have shown how single or overlapping spatial expression of claudins can generate unique domains of ion permeability, thus creating gradients in the microenvironment that can guide patterning in chick embryos. Claudins assemble with other proteins to form complexes and, in contrast to what has been observed for integrins and cadherins, these complexes may include Ephs or ephrins. Ephs and ephrins are widely expressed during embryogenesis, especially during the establishment of tissue boundaries in vertebrates, and guide cell segregation primarily through repulsive interactions. In Xenopus, EphA7 was shown to be present as both a full-length membrane receptor and a soluble (truncated) molecule (sEphA7). These two forms play different roles in pronephros development *in vivo*, and their interaction with the tight junction protein claudin6 has different outcomes: Sun and collaborators (Sun et al., 2018) reported physical interactions in *cis* between full-length EphA7 and claudin6, in which EphA7 binds and phosphorylates claudin6 thus directly regulating its localization and distribution at the cell membrane, in an ephrin-independent manner. sEphA7 stabilizes claudin6 at the cell membrane by antagonizing the action of full-length EphA7. The authors speculated that one possible mechanism could be that sEphA7 may dimerize with its full-length counterpart, acting in a dominant-negative fashion by preventing *trans*-autophosphorylation and subsequent regulation of claudin6 distribution at the cell surface, *in vitro* and *in vivo*.

Tanaka et al. (2005a) showed *cis* activation of ephrin-B1 via claudins (i.e., formation of the complex with claudin1 and claudin4) which led to enhanced ephrin-B1 phosphorylation and cell-cell adhesion in a claudin-mediated fashion in epithelial cells. In colon cancer, EphA2 interacts with claudin4 to regulate cell adhesion via *cis* interactions leading to EphA2-dependent phosphorylation of claudin4; phosphorylated claudin is not recruited to the tight junction and, as a consequence, the authors observed increased paracellular permeability, a factor that promotes cancer aggressiveness (Tanaka et al., 2005b).

#### 3.4.4 Other Membrane Proteins

VEGFs and their receptors VEGFRs are key molecules involved in physiological processes and in cancer due to their central role in angiogenesis. They accomplish their action by forming ternary protein complexes with additional molecules, which can include EphA2 in the context of cancer. IP and PLA have demonstrated a physical interaction between EphA2 and VEGFR2 in non-small cell lung carcinoma cells which promotes tumor cell migration and cancer invasiveness; when phosphorylation of serine (S) 897 of EphA2 is prevented these activities are impaired. Conversely, an increase in tumor cell motility and invasion was observed upon S897 phosphorylation of EphA2 in combination with anti-VEGFR2 treatment (Volz et al., 2020).

#### 3.4.5 Outside and Inside the Cell

Adhesion signaling begins at the plasma membrane level then is transduced inside the cell via intracellular signaling molecules including metalloproteases, Src family kinases, and the small G-protein Rho. Meltrin β (ADAM19) is a membrane metalloprotease that interacts in *cis* with EphA4 through its ectodomain (Figures 1C, 3B). This interaction is of particular interest for the formation of neuromuscular junctions (NMJs). Yumoto and collaborators (Yumoto et al., 2008) describe a role for meltrin β in the development of NMJs via binding to EphA4 and subsequent stabilization of the Eph-ephrin complex between EphA4 and ephrin-A5, acting as regulators of cell repulsion. This is triggered by internalization of the Eph-ephrin complex; meltrin β regulates the endocytosis of EphA4-ephrin-A5 complex, thus tuning cell repulsion and the formation of NMJs during muscle and nervous system development. Other ADAM family members, such as ADAM10 (Kaplan et al., 2018), also regulate cell adhesion and cell migration, however as per the most of the crosstalk with integrins and cadherins the interaction with the ephrin system is biochemical but not physical.

As discussed earlier, Src is activated upon integrin engagement to promote integrin-mediated adhesion. EphA2-ephrin-A1 signaling has been demonstrated to activate Src to regulate focal adhesion assembly and cell motility (Figure 3A) (Webb et al., 2004); after binding, EphA2-ephrin-A1 complexes accumulate in clusters creating high ephrin density sites in close proximity to FAs. It is at these regions on the plasma membrane that Src is recruited. Chen et al. (2018) showed that the interaction between Src-EphA2-ephrin-A1 is crucial for the translocation of Src to FAs, which is involved in adhesion turnover. While Src recruitment to FAs is ephrin-mediated in this context, the effect of EphA2-ephrin-A1 signaling on FA dynamics occurs in a Src-dependent manner. These findings are of particular interest to highlight the effect of the ephrin system on integrin signaling promoting adhesion and migration; Ephs and ephrins do not directly interact with integrins to regulate these cellular processes but rather associate with downstream effectors that participate in integrin-dependent pathways such as Src.

EphA2 also promotes adhesion formation and cell migration through the activation of RhoA, another cytoplasmic molecule that plays a key role in cell adhesion through FAK (Parri et al., 2009), likely via the formation of a protein complex including Src. However, to the best of our knowledge, no evidence for direct RhoA-Eph/ephrin interactions has been reported. In contrast, ephrin-B1 has been shown to regulate cell-cell adhesion and cell motility by interacting with connector enhancer of kinase suppressor of Ras1 (CNK1) and RhoGDI1 in a Src-mediated manner (Figure 3C). Cho et al. (2018) showed that non-canonical (receptor-independent) ephrin-B1 signaling modulates cell adhesion and migration. Upon Src-dependent activation, CNK1 interacts in *cis* with ephrin-B1 and this protein complex is then primed to recruit other molecules such as RhoGDI1 through direct binding of an 8-amino acid (aa) region in the intracellular tail of ephrin-B1 to RhoGDI1 (Figure 1D), thus promoting cell migration. Therefore, the ephrin system may indirectly activate RhoA signaling via engaging RhoGDI1 in a Src-dependent fashion in the context of cancer.

Another member of the Ras superfamily whose activity interlocks with ephrin pathways is Rab35-GTPase activating protein (GAP). Rab and its GAP TBC1d24 inhibit cell motility during neurodevelopment. The function of TBC1d24 must be highly regulated to provide timely migration of cranial neural crest cells to achieve precise development. One of the molecules physically interacting with GAP is ephrin-B2 which, together with its receptor EphB4, directs cell migration during neurodevelopment. Yoon et al. (2018) showed that a TBC1d24-ephrin-B2 complex is able to modulate cell migration of neural crest cells, but interestingly it requires ephrin-B2 to remain in a non-phosphorylated state: phosphorylation of ephrin-B2 causes a decrease in the interaction with TBC1d24 and impairs the migration of cranial neural crest cells. In addition, this complex was demonstrated to also affect cell migration via the regulation of the expression of E-cadherin. This highlights another case in which an ephrin modulates cellular processes via physical interaction with molecules other than its receptor.

### 3.5 Contribution of Materials Science to the Study of Cell Adhesion and Migration

Materials science is gaining interest in developmental biology, especially in the study of cellular processes where physical stimuli and the biochemical composition of the microenvironment are driving factors (such as cell adhesion). The development of tools specifically designed to address cell adhesion, including micro- and nano-patterning and the design of microfluidic systems, have been among the most popular strategies exploited so far. These approaches enable the study of attachment and detachment events at the single cell or cell population levels, providing insights on the mechanics of cell adhesion to a substrate and how cells make contact with each other by measuring parameters such as adhesion strength (for instance, by assessing micro-pillar displacement upon cell adhesion).

Micro-patterning is widely used for adhesion force measurements, especially via micro-fabricated elastomeric post arrays. Biocompatible substrates can be designed to present a surface characterized by elastomeric pillars (different substrates can have different stiffness and size of these pillars) and used in cell culture to measure cell adhesion force based on pillar displacement (Califano and Reinhart-King, 2010; D’Arcangelo and McGuigan, 2015). Another approach of patterning to cell adhesion is nano-patterning with the deposition of either biocompatible materials or adhesion molecules on a biopolymer support. This strategy allows the experimenter to spatially control cell adhesion and manipulate the substrate surface properties in order to tune cellular responses (e.g., cytoskeleton rearrangements or modulation of transcription) to different biochemical signals (Spatz and Geiger, 2007; Díaz Lantada et al., 2020). Micro-patterning can also be exploited to investigate cellular responses to microenvironment topography in terms of cell migration. Micro- and nano-patterns can mimic the *in vivo* topography of tissues, such as cell and cell protrusion alignment in the nervous system, to create directionality cues (Nedjari et al., 2017; Wang et al., 2019; Cun and Hosta-Rigau, 2020). The effects of micro-patterning on cell architecture and functions have been extensively reviewed by Théry (Théry, 2010).

Microfluidics represents the most versatile tool for modifying and manipulating cell adhesion *in vitro*. A wide range of reports have described microfluidic systems to study both attachment and detachment events in either a single cell or a cell population. A detailed list of microfluidic applications to address cell adhesion has reported in a review by Khalili and Ahmad (2015), including platforms to simulate the human vascular system for biomedical analysis. The greatest advantages of microfluidic devices are the ability not only to tune the fluid flow and its 3D design, but also to obtain miniaturized and standardized flow chambers for cell culture. Additionally, the possibility to create different layers and compartments also allows the study of cell adhesion in parallel with other cellular events, such as cell migration. The use of these *in vitro* tools could overcome some of the drawbacks of standard *in vitro* approaches and *in vivo* studies (absence of flow and high costs, respectively). Three-dimensional cultures in combination with bioinformatics may represent a compromise between *in vitro* and *in vivo*, and, due to their robustness and reproducibility, are acquiring increasing interest in fields of research like the emerging field of mechanobiology. Considering the strict correlation between cell adhesion and migration and mechanical forces, a materials science approach has promise for providing a better understanding of these cellular mechanisms, giving the experimenter control of parameters such as substrate elasticity and stiffness as well as geometry and surface chemistry.

## 4 Cell Sensing: Cell-Microenvironment Crosstalk

### 4.1 The Physics of Cell Sensing

Cell sensing is the ability of cells, especially those cells building highly aligned and organized tissues (e.g., neurons and muscles), to read and adapt to the surrounding topography and mechanical cues. Proteins primarily associated with cell sensing are CAMs, such as cadherins, and small GTPases of the Rho family (Mayor and Carmona-Fontaine, 2010). Chemotaxis and force sensing work together to achieve cell adhesion and cell polarity, and ultimately can lead to directed cell migration. Cell adhesion and directional migration can be influenced by forces directly applied to cells (e.g., shear stress) and/or matrix stiffness and elasticity (Espina et al., 2021; Yin et al., 2021). In addition to mechanical stimuli, cells can redirect each other to generate an organized tissue structure. An example is the alignment of neuronal cells on anisotropic cell culture substrates (Tonazzini et al., 2014) that can mimic cell organization of neurites *in vivo* (Hatten, 1990; Hoffman-Kim et al., 2010). The process through which a cell, such as a neuron, reads the composition of its surrounding environment (e.g., ECM heterogeneity), adheres, and polarizes to directionally migrate was defined as “contact guidance” by Paul Weiss in the early 40s (Weiss, 1945). The formation of adhesion sites (i.e., focal complexes and FAs) and contact guidance through sensing of the microenvironment geometry and rigidity promote optimal function of cells in adult tissues (Kolahi and Mofrad, 2010; Dufort et al., 2011) as well as correct patterning during embryogenesis (Kolahi and Mofrad, 2010; Agarwal and Zaidel-Bar, 2021).

### 4.2 How the Ephrin System Tunes Cell Sensing: Protein Complexes and Microenvironment Reading

Adhesion molecules play a pivotal role in the sensing of environment topography, rigidity, and other external physical cues (e.g., shear stress). Among the cell adhesion molecules that have been shown to be able to respond to and transduce mechanical stimuli are cadherins and integrins, which have been linked to cellular changes in response to force (Maître and Heisenberg, 2013; Kechagia et al., 2019). Both cadherins and integrins act as surface mechanoreceptors and transduce external physical forces generated by interactions with other cells or with the ECM, to initiate mechanostransduction signaling cascades ultimately leading to gene expression changes (Schwartz and DeSimone, 2008). Downstream molecules involved in mechanotransduction are often small G-proteins of the Ras superfamily such as RhoA and Rac1 (Nelson et al., 2004; Theveneau et al., 2010), whose role in mediating cell adhesion and migration has been discussed above. Their close interaction with the cytoskeleton also makes them candidate molecules for cell sensing studies, as they promote the formation of cytoskeletal structures, such as stress fibers, in response to mechanical cues.

**FIGURE 4:**
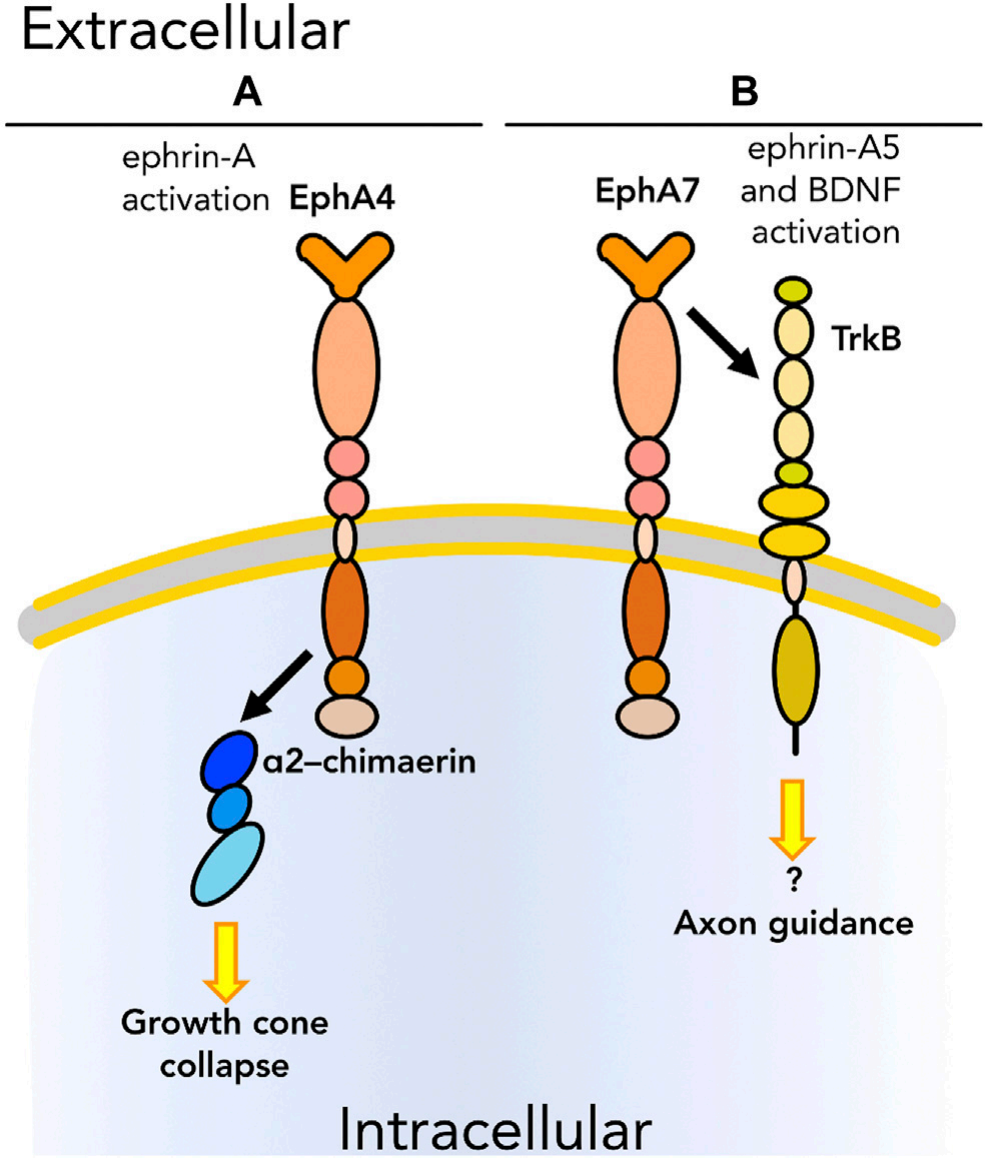
Graphical representation of *cis* physical interactions occurring between Ephs and other molecules to promote or inhibit the ability of cells to “read” the surrounding environment: cell sensing. **(A)** EphA4 interacts with α2–chimaerin, that results in growth cone collapse. **(B)** EphA7 regulates axon guidance by interacting with TrkB upon, respectively, ephrin-A5 and BDNF binding. As we know only some of the downstream pathways, unknown downstream effectors are listed with a question mark. Receptors are depicted as single chains for simplicity. References are listed in Table 1.

Aside from their well-described roles in directing cell migration (primarily through repulsive interactions), Ephs and ephrins also participate in guidance signaling via modulation of other adhesion and migration molecules (Table 1, blue section; Figure 4). Although as noted earlier Ephs and ephrins have only rarely been shown to physically interact with integrins and cadherins, physical association with members of the Rho family has been observed in multiple biological contexts. In the case of neuronal pathfinding, there is evidence in the literature of physical interaction between EphA4 and members of the Rho family as well as other proteins involved in Rho pathways including α2–chimaerin (Figure 4A), a Rac1–activating protein, during growth cone extension (Shi et al., 2007). Growth cones are structures fundamental for the establishment of neuronal connections. Through these structures at the tips of neuronal protrusions, neurons are able to sense the surrounding environment and find the route to interact with their targets to form synapses. Axon guidance is thus a biological and mechanical process leading to the formation of a functional nervous system. The interaction between EphA4 and α2–chimaerin was shown to be crucial for Eph–dependent growth cone collapse. Shi et al. (2007) demonstrated that the two proteins physically interact in *cis* upon ephrin-A activation of EphA4. EphA4 then activates α2–chimaerin which leads to activation of RhoA and inhibition of Rac1, limiting cell adhesion. In parallel, Wegmeyer et al. (2007) also demonstrated the role of EphA4-α2–chimaerin in regulating axon guidance in neurons. Another interaction in *cis* between the ephrin system and neuro-specific molecules was reported by Fitzgerald et al. (2008). EphA7 and BDNF receptor TrkB were demonstrated to physically interact upon activation after binding of ephrin-A5 and BDNF, respectively, and to regulate axon guidance (Figure 4B) in the development of the visual system. As the tissue is developing its organized structure, the ephrin system in cooperation with BDNF mediates reading of the nascent topography and subsequent growth cone formation.

To our knowledge, cell sensing has been studied almost exclusively in nervous system models. Other tissues showing a cellular organization that leads to an aligned topography, such as the skeletal muscle, or other tissue maintenance mechanisms (e.g., wound healing) might be valuable models to study the importance of in *cis* protein complexes able to tune cell sensing.

## 5 Conclusion

Cell signaling at the plasma membrane is rarely as simple as the linear interaction between ligand and receptor it is often depicted as; the formation of heterotypic protein complexes is key for successful and appropriate transmission of signaling information. Over the past two decades, the study of Ephs and ephrins has been extended from their canonical roles in development to their involvement in mediating cellular processes in adult tissues and cancer. Their ubiquitous expression and promiscuity of binding contribute to the broad range of potential signaling outcomes this family participates in. Other signaling pathway interactions, such as Wnt or FGF, are well-known to incorporate “secondary” signaling molecules that interact physically and/or biochemically with key proteins [i.e., transmembrane co-receptors Lgr and LRP (Niehrs, 2012) or heparan sulfate proteoglycans (Allen and Rapraeger, 2003)]. We suggest that equally important but less well-described roles for Ephs and ephrins, acting as either the primary signaling receptor or as crucial members of ternary complexes, are involved in a wide range of cellular mechanisms, from cell proliferation and differentiation, to adhesion and migration, to cell sensing. In particular, interactions of Eph/ephrins with other molecules at the plasma membrane in *cis* (“horizontal” interactions) are not yet as well-characterized as those involving ligands originating from a different cell or downstream effectors (“vertical” interactions). We are hopeful that additional insights gained in future studies will lead to not only an improved understanding of these critical signaling pathways, but also to novel translational applications in human health.
